# Cardiovascular Toxicity of Multi-Tyrosine Kinase Inhibitors in Advanced Solid Tumors: A Population-Based Observational Study

**DOI:** 10.1371/journal.pone.0122735

**Published:** 2015-03-27

**Authors:** Amirrtha Srikanthan, Josee-Lyne Ethier, Alberto Ocana, Bostjan Seruga, Monika K. Krzyzanowska, Eitan Amir

**Affiliations:** 1 Division of Medical Oncology and Hematology, Princess Margaret Cancer Centre and Department of Medicine, University of Toronto, Toronto, Ontario, Canada; 2 Institute of Health Policy, Management and Evaluation, University of Toronto, Toronto, Ontario, Canada; 3 Medical Oncology Department and Translational Research Unit, Albacete University Hospital, Edificio de Investigación, Calle Francisco Javier de Moya, Albacete, Spain; 4 Department of Medical Oncology, Institute of Oncology Ljubljana, Ljubljana, Slovenia; Seoul National University, KOREA, REPUBLIC OF

## Abstract

**Background:**

Treatment with small molecule tyrosine kinase inhibitors (TKIs) has improved survival in many cancers, yet has been associated with an increased risk of adverse events. Warnings of cardiovascular events are common in drug labels of many TKIs. Despite these warnings, cardiovascular toxicity of patients treated with TKIs remains unclear. Here, we evaluate the cardiovascular outcomes of advanced cancer patients treated with small molecule tyrosine kinase inhibitors.

**Methods:**

A population based cohort study was undertaken involving adults aged >18 years in Ontario, Canada, diagnosed with any advanced malignancy between 2006 and 2012. Data were extracted from linked administrative governmental databases. Adults with advanced cancer receiving TKIs were identified and followed throughout the time period. The main outcomes of interest were rates of hospitalization for ischemic heart disease (acute myocardial infarction and angina) or cerebrovascular accidents and death.

**Results:**

1642 patients with a mean age of 62.5 years were studied; 1046 were treated with erlotinib, 166 with sorafenib and 430 with sunitinib. Over the 380 day median follow-up period (range 6-1970 days), 1.1% of all patients had ischemic heart events, 0.7% had cerebrovascular accidents and 72.1% died. Rates of cardiovascular events were similar to age and gender-matched individuals without cancer. In a subgroup analysis of treatment patients with a prior history of ischemic heart disease, 3.3% had ischemic heart events while 1.2% had cerebrovascular accidents.

**Conclusions:**

TKIs do not appear to increase the cause-specific hazard of ischemic heart disease and cerebrovascular accidents compared to age and gender-matched individuals without advanced cancer.

## Background

More than 90 tyrosine kinases have been shown to be critical to malignant transformation and tumor angiogenesis [1, 2]. Tyrosine kinase inhibitors (TKIs), which can target both receptor and cytoplasmic kinases [3] can improve cancer outcomes by exploiting activation of kinases in cancer cells [4]. A number of different TKIs have been studied and approved for use in both solid tumors and haematological malignancies [5–12]. Commonly used TKIs include erlotinib targeting epidermal growth factor receptor (EGFR), and sorafenib and sunitinib targeting mainly vascular endothelial growth factor receptor (VEGRF) and platelet-derived growth factor receptor (PDGFR) [13]. In addition to improved outcomes, TKIs are also relatively easy to administer [14].

Despite this targeted intent, TKIs usually affect multiple kinases [15, 16] and impact the function of non-malignant cells with resultant on- and off-target toxicities [13]. On-target toxicities, such as hypertension from VEGFR inhibitors, are due to class effects and are difficult to prevent [13]. Off-target toxicities occur when unintended targets are inhibited by the drug due to similarities with the intended target [13]. Both on- and off-target adverse events are described in clinical trials [17]. Non-cardiac toxicities from TKIs include skin toxicity, diarrhea, mucositis, pneumonitis and electrolyte abnormalities [13].

Hypertension, congestive heart failure, left ventricular systolic dysfunction and QT prolongation are common adverse cardiac toxicities associated with TKIs [3, 18–23]. Cardiovascular events, such as cardiac ischemia, myocardial infarction and cerebrovascular accidents, have also emerged as concerning toxicities such that drug label warnings have been issued for TKIs [24–26]. Given the broad patient populations eligible for TKI treatment, the improved survival seen with targeted agents [5–12], and the prevalence of cardiovascular disease in the population [27], recognition, management and prevention of TKI related cardiovascular events have emerged as important [19]. The development of strategies and guidelines to assess this emerging issue [21, 28, 29] and recommendations for cardiac safety monitoring of patients undergoing TKI treatment in clinical practice [30–32] have therefore been recommended.

Clinical trial reports of cardiovascular toxicity are limited by inadequate power and are known to underrepresent at risk patients such as older individuals or those with significant comorbidities [33]. Patients enrolled in RCTs are highly selected and likely not representative of patients treated in general practice [34]. Efficacy and toxicity outcomes have been shown to be different between patients treated on and off clinical trials even when treated at the same institution at the same time [35]. Therefore, an assessment of the potential cardiovascular toxicities of TKIs in a population of unselected cancer patients is desirable.

Here, we report on a population based observational cohort study to assess the rates of cardiovascular and cerebrovascular outcomes and death among cancer patients receiving TKIs. We hypothesized that, compared to the general population, cardiovascular events and cerebrovascular accidents would be more prevalent in a population of patients with advanced cancer receiving TKIs, particularly among individuals with a prior history of ischemic heart disease (IHD).

## Materials and Methods

### Data Sources

The Ontario Health Insurance Plan (OHIP) is a publically funded health insurance program providing universal coverage for medically necessary care in Ontario, Canada—Canada’s most populous province with approximately 13.5 million residents [36]. Each resident is assigned a unique Ontario Health Insurance Number (OHIN), which was used to link multiple administrative health databases. Databases and data sets were held securely in a linked, de-identified form and analyzed through the Institute for Clinical Evaluative Sciences (ICES).

The Ontario Cancer Registry (OCR) is a passive registry of invasive cancer diagnoses of Ontario residents from 1964 onwards [37, 38]. This registry was used to identify patients with oncological diagnoses. The Ontario Drug Benefit (ODB) program reimburses prescription medication for all Ontario residents ≥ 65 years old. Individuals between 18–64 years of age are eligible for ODB support only if requiring long-term care, home care, governmental financial assistance, disability support or financial assistance (defined as high prescription drug costs relative to income) [39]. The ODB was used to determine exposure to TKI medications and exposure to cardiac medications prescribed before the first TKI prescription. TKI medications were prescribed in line with ODB Exceptional Access Program (EAP) eligibility for these drugs [40].

Oncologic drug availability is a multi-step process in Canada. After federal Health Canada approval, the national pan-Canadian Oncology Drug Review (pCODR) evaluates a new cancer agent’s efficacy and cost-effectiveness. A recommendation regarding funding the new agent is then issued. Individual provinces then review pCODR recommendations and manufacturer requests. Individual provincial funding decisions are made after consideration of expert reviews, governmental budgets and the public interest [41]. Once provincially approved by the Ontario Ministry of Health and Long-Term Care, an intravenous drug is available throughout Ontario through the New Drug Funding Program (NDFP). Oral agents are funded through the ODB, private insurance or self-pay mechanisms [42]. Prices of anti-cancer agents are negotiated privately between provinces and manufacturers and are details of agreements are not generally available publically.

The NDFP was used to identify individuals who received systemic treatment prior to or after TKI initiation. The Canadian Institute for Health Information Discharge Abstract Database (CIHI-DAD) holds a record of all discharges from acute care hospitals in Ontario. The National Ambulatory Care Reporting System (NACRS) provides a record of hospital and community based emergency and ambulatory care. The OHIP database also provides additional data of physician services from billing claims.

Baseline comorbidities were extracted from CIHI-DAD and NACRS using corresponding International Classification of Diseases codes (ICD10) in the 10 years preceding the index case. Baseline hypertension, IHD, congestive heart failure and diabetes were identified using validated algorithms. These algorithms use both in- and out-of-hospital diagnostic and billing codes [43–46].

The Ontario Registered Persons Database (ORPD) provided demographic data such as vital status, postal code and date of death. Cause of death was not available in the database. Canadian Census data was used to establish median household neighbourhood income, which was used as a surrogate for socioeconomic status [47]. Limitations of available databases prevented determination of when death occurred relative to TKI treatment completion.

### Study Design

The OCR was used to identify adults ≥ 18 years old from January 1, 2006 to September 30, 2012 with a first documented diagnosis of cancer. Prescription drug information was available for all adults ≥ 65 years old and limited for individuals between 18–64 years of age due to the aforementioned provincial criteria.

The ODB was used to identify all adults who were dispensed a TKI prescription at any point after their date of diagnosis. All individuals with any exposure to a TKI were initially included. The control group comprised all age and gender-matched individuals without cancer in the Ontario population during the time of interest. The control group was derived from the general population and was compared to the treatment group with respect to additional comorbidities. Drugs with fewer than 50 exposed patients were excluded from the analysis to reduce heterogeneity.

The impact of treatment on the cause specific hazard of IHD and cerebrovascular disease and on the hazard of death was evaluated. Outcomes were determined by identifying hospitalizations with a most responsible diagnosis of acute myocardial infarction (AMI), angina (ICD10 I20-I22 or I24) or cerebrovascular accident (ICD10 I60-I69 or G45). The outcome of IHD was defined as a composite of AMI and angina.

### Statistical Analysis

Descriptive statistical analyses were utilized and frequency of occurrence and percentage was calculated for each of the independent variables. Continuous baseline variables were compared between the treatment and control group using Wilcoxon Rank Sum test. Categorical baseline variables were compared using the χ^2^ statistic. Time-to-event analyses were performed using the control population as the reference for each of the outcomes of interest. Time-to-event was defined as the time from first prescription of TKI to the event of interest. Hazard ratios (HR) and 95% confidence intervals (CI) were calculated. Pre-stratified subgroup analyses were performed based on the presence of previously diagnosed IHD. Patients were censored if an event of interest did not occur prior to September 30, 2012 (the end of follow-up). In the analysis of cause specific hazard for IHD or cerebrovascular disease, patients were censored at death or if they received systemic therapy or radiation during the year after TKI initiation. Kaplan-Meier survival curves were plotted to illustrate overall survival free of death, free of IHD and free of cerebrovascular accidents. All analyses were performed using SAS software, version 9.2 (SAS Institute Inc., Cary, NC). Statistical significance was defined using a two-tailed p-value of <0.05. Data cells involving ≤ 5 patients were not included in keeping with ICES’ privacy regulations.

### Ethics Statement

The ICES review board and privacy office approved this study prior to commencement. Consent was not obtained from individual patients; however, all patient information was anonymized and de-identified by ICES prior to receipt by the investigators for analysis.

## Results

Seven TKIs were publically funded and in use between 2006 and 2012 in Ontario. Data for these drugs comprised 1046 patients treated with erlotinib, 430 with sunitinib, 166 with sorafenib, 46 with gefitinib, 11 with everolimus, 9 with temsirolimus and 5 with lapatinib. Data for gefitinib, everolimus, temsirolimus and lapatinib were excluded and consequently, a cohort of 1642 patients exposed to erlotinib, sunitinib and sorafenib were included in the analysis. These treatment patients were compared to 128,415 age and gender matched individuals without cancer who served as controls. The mean age of the TKI-treated patients was 65.2 years and 338 (20.6%) were identified as having baseline IHD, including 99 individuals (6.0%) who had a previous AMI (Table 1).

**Table 1:** Baseline Characteristics of the TKI-Treated Patients and Control Group.

**Variable**	Value	Treatment	Control	p-value
		N = 1,642	N = 128,415	
****Age****	Mean ± Standard Deviation	65.23 ± 10.57	65.40 ± 13.39	0.611
	< 65	645 (39.3%)	58,772 (45.8%)	<0.001
	≥ 65	997 (60.7%)	69,643 (54.2%)	
	< 39	25 (1.5%)	N/A*	
	40–64	620 (37.8%)	N/A*	
	65–74	691 (42.1%)	N/A*	
	75+	306 (18.6%)	N/A*	
****Sex****	Female	722 (44.0%)	87,571 (68.2%)	<0.001
	Male	920 (56.0%)	40,844 (31.8%)	
****Tumor Type****	Non-small cell lung cancer	1046 (63.7%)	N/A	
	Renal cell carcinoma	516 (31.4%)	N/A	
	Hepatocellular carcinoma	80 (4.9%)	N/A	
****Cardiac Events 10 Years Before Index Date****	Acute Myocardial Infarction	99 (6.0%)	7,042 (5.5%)	0.335
	Angina	45 (2.7%)	5,028 (3.9%)	0.015
	Congestive Heart Failure	118 (7.2%)	11,055 (8.6%)	0.041
	Coronary Angiography	136 (8.3%)	9,584 (7.5%)	0.21
	Percutaneous Coronary Intervention	45 (2.7%)	3,110 (2.4%)	0.404
	Coronary Artery Bypass Graft	25 (1.5%)	2,383 (1.9%)	0.32
	Cerebrovascular Disease	33 (2.0%)	4,974 (3.9%)	<0.001
	Peripheral Vascular Disease	111 (6.8%)	7,234 (5.6%)	0.049
	Ischemic Heart Disease	338 (20.6%)	23,104 (18.0%)	0.007
	Dyslipidemia	580 (35.3%)	37,028 (28.8%)	<0.001
	Chronic Dialysis	≤5 (0.3%)	587 (0.5%)	0.361
	Venous Thromboembolism	143 (8.7%)	1,913 (1.5%)	<0.001
	Renal Disease	94 (5.7%)	5,882 (4.6%)	0.028
****Cancer Treatment Before Index Date****	Chemotherapy	1,230 (74.9%)	3,377 (2.6%)	<0.001
	Radiation	837 (51.0%)	2,236 (1.7%)	<0.001
****Baseline Medications****	ACE Inhibitors	370 (22.5%)	26,171 (20.4%)	0.031
	ARBs	192 (11.7%)	10,970 (8.5%)	<0.001
	Aspirin	48 (2.9%)	5,331 (4.2%)	0.013
	Thienopyridene Derivatives	42 (2.6%)	3,921 (3.1%)	0.246
	Beta Blockers	310 (18.9%)	20,335 (15.8%)	<0.001
	Calcium Channel Blockers	368 (22.4%)	21,846 (17.0%)	<0.001
	Digoxin	35 (2.1%)	3,273 (2.5%)	0.286
	Anti-Dyslipidemia Medications	532 (32.4%)	34,394 (26.8%)	<0.001
	Aldosterone Antagonists	42 (2.6%)	2,806 (2.2%)	0.305
	Loop Diuretics	197 (12.0%)	11,771 (9.2%)	<0.001
	Other Diuretics	211 (12.9%)	15,542 (12.1%)	0.356
	Statins	510 (31.1%)	32,933 (25.6%)	<0.001
	Oral Hypoglycemics	248 (15.1%)	11,972 (9.3%)	<0.001
	Insulins	61 (3.7%)	3,339 (2.6%)	0.005
	Warfarin	100 (6.1%)	6,668 (5.2%)	0.104
	Low Molecular Weight Heparin	177 (10.8%)	896 (0.7%)	<0.001
	Nitrates	79 (4.8%)	7,869 (6.1%)	0.027
	Non-Steroidal Anti-Inflammatory Drugs	318 (19.4%)	18,541 (14.4%)	<0.001
****Physician Visits in Past Year****	Primary care provider visits in past year	16.13 ± 14.08	9.80 ± 9.40	<0.001
	Specialist visits/consults in past year	31.46 ± 16.51	9.36 ± 9.94	<0.001
	Total number of physician visits in past year	48.99 ± 22.83	19.31 ± 15.87	<0.001
****Drug Name****	Erlotinib	1046 (63.7%)	N/A	
	Sorafenib	166 (10.1%)	N/A	
	Sunitinib	430 (26.2%)	N/A	

ACE, Angiotensin Converting Enzyme; ARBs, Angiotensin II Receptor Blockers; N/A*, Not Available; N/A, Not Applicable; TKI, tyrosine kinase inhibitor

### Baseline Comparison of TKI-treated Individuals to Individuals Without Cancer

Baseline characteristics of the treatment and control group are shown in Table 1. The control group of age and gender-matched individuals without cancer was of similar age (65.4 versus 65.2, p = 0.61), but less likely to have prior IHD (18.0% versus 20.6%, p = 0.007), venous thromboembolism (1.5% versus 8.7%, p<0.001), renal disease (4.6% versus 5.7%, p = 0.028), and cardiac medication usage (angiotensin converting enzyme inhibitors, 20.4% versus 22.5%, p = 0.031; angiotensin receptor blockers, 8.5% versus 11.7%, p<0.001; beta-blockers, 15.8% versus 18.9%, p<0.001; calcium channel blockers, 17.0% versus 22.4%, p<0.001 and anti-dyslipidemia medications, 26.8% versus 32.4%, p<0.001). Baseline diagnosis of diabetes was not available; however, baseline usage of both oral hypoglycaemic agents (9.3% versus 15.1%, p<0.001) and insulin (2.6% versus 3.7%, p = 0.005) was less common among the control group. Usage of aspirin was greater among control patients (2.9% vs. 4.2%, p = 0.013). All TKI-treated patients had a stage IV cancer diagnosis, in keeping with ODB EAP eligibility criteria [40].

### Ischemic Cardiac and Cerebrovascular Outcomes and Death

Within the 380-day median follow-up period after TKI therapy initiation, 18 (1.1%) of the 1642 treatment patients developed an ischemic heart disease event requiring hospitalization, 11 (0.7%) developed a cerebrovascular accident requiring hospitalization and 1184 (72.1%) individuals died. Of the 18 cases of ischemic heart disease, 11 occurred in erlotinib treated patients, 5 in sunitinib treated patients and 2 in sorafenib treated patients. Of the 11 cases of cerebrovascular accidents, 8 occurred in erlotinib treated patients, 2 in sunitinib treated patients and 1 in sorafenib treated patients. These proportions closely mirrored the relative frequency of drug use in the population.

**Fig 1:**
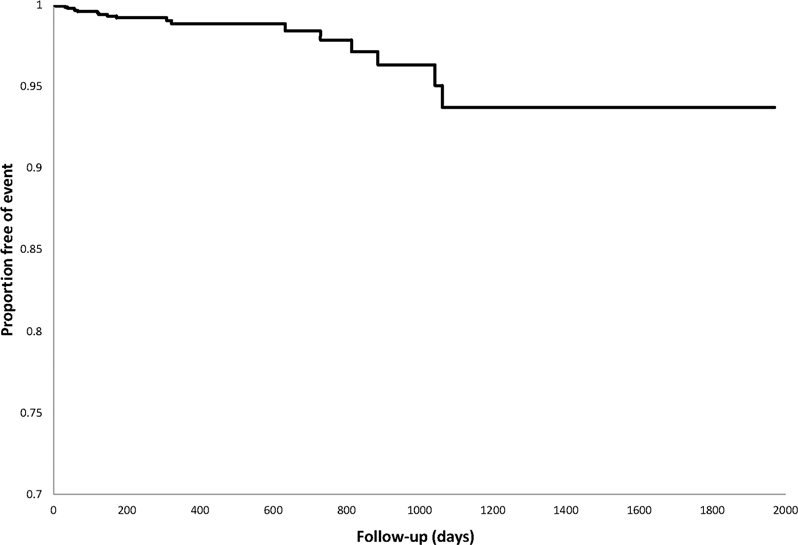
Kaplan Meier curve for time to cardiovascular event in tyrosine kinase inhibitor (TKI)-treated group.

**Fig 2:**
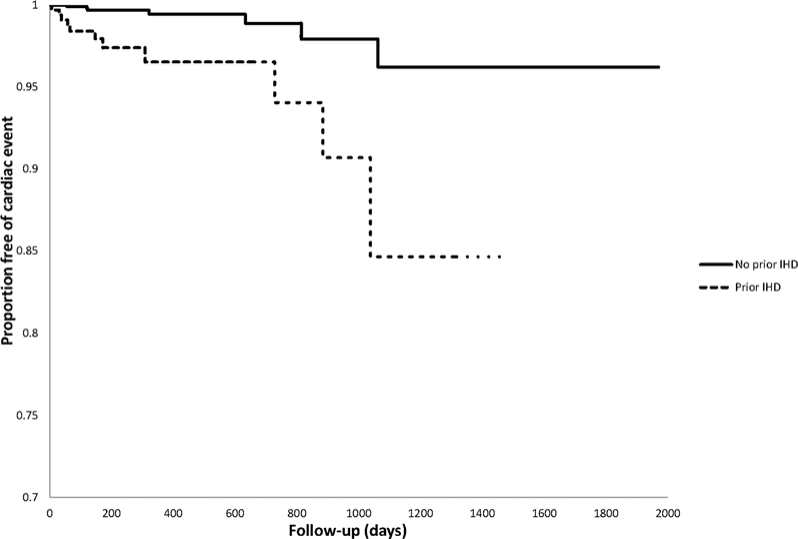
Kaplan Meier curves for time to cardiovascular event in tyrosine kinase inhibitor (TKI)-treated group based on history of ischemic heart disease (IHD).

Cardiovascular events predominantly occurred late in follow-up (see Fig. 1). Of the 338 patients with baseline IHD, 11 (3.3%) had ischemic heart events during follow-up while 4 (1.2%) had cerebrovascular accidents and 245 (72.5%) died. Of the 1304 patients without baseline IHD, 7 (0.5%) had ischemic heart events, 7 (0.5%) had cerebrovascular accidents and 939 (72.0%) died. A comparison of time to cardiovascular event between patients with and without prior IHD is shown in Fig. 2. Compared to those without prior IHD, there was a numerical, but non-significantly higher hazard of cardiovascular events in those with prior IHD (HR 1.59, 95% CI 0.76–3.33, p = 0.22). Power to detect differences between these groups was low (20.3% assuming alpha = 0.05).

### Comparison of Outcomes to Individuals Without Cancer

**Fig 3:**
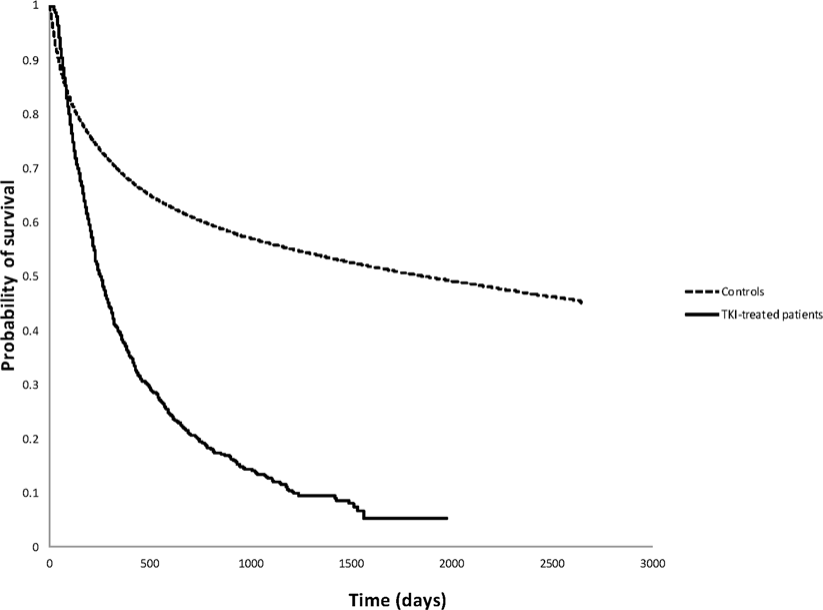
Kaplan Meier curves for overall survival in tyrosine kinase inhibitor (TKI)-treated and control groups.

Compared to age and gender-matched non-cancer patients, patients exposed to TKI had similar rates of IHD and cerebrovascular accidents (Table 2), but a significantly higher hazard of death (Fig. 3). Results were similar for both the whole study population and the subgroup with IHD (Table 2). Due to small event numbers, additional subgroup analyses based on duration of treatment or type of TKI were not undertaken.

**Table 2:** Ischemic Cardiac and Cerebrovascular Outcomes and Death—Compared to the Control Group.

Variable	Number (%)	HR	95% CI	p-value
**1. Entire Population**				
Mortality	1184 (72.1)	1.73	1.63–1.84	<0.0001
Cerebrovascular Accidents	11 (0.7)	0.62	0.34–1.12	0.11
Ischemic Heart Events	18 (1.1)	0.82	0.52–1.30	0.4
**2. No Prior Ischemic Heart Disease**				
Mortality	939 (72)	1.84	1.73–1.97	<0.0001
Cerebrovascular Accidents	7 (0.5)	0.54	0.26–1.14	0.10
Ischemic Heart Events	7 (0.5)	0.64	0.30–1.34	0.24
**3. Prior Ischemic Heart Disease**				
Mortality	245 (72.5)	1.38	1.22–1.57	<0.0001
Cerebrovascular Accidents	4 (1.2)	0.80	0.30–2.14	0.65
Ischemic Heart Events	11 (3.3)	1.02	0.56–1.85	0.94

CI, confidence interval; HR, hazard ratio; Ischemic Heart Events (includes both Acute Myocardial Infarctions and Angina)

## Discussion

While data are available to inform about TKI-induced cardiac toxicity among patients treated in clinical trials, less is known about such toxicities in patients treated in routine clinical practice. This population-based study of all patients treated in Ontario, Canada demonstrates that TKI use among advanced cancer patients did not show significantly higher rates of cardiovascular adverse events relative to the general population. Furthermore, those events which did occur appeared to be more frequent later in follow-up. As expected, in the stratified analyses, a trend for higher rates of ischemic heart disease and cerebrovascular accidents was seen among individuals with a history of prior IHD. Despite TKI-treated patients having higher rates of baseline cardiac comorbidities and usage of cardiac medications than the general population, rates of cardiac events were low overall, while death events (presumably from the underlying malignancy) were frequent.

Although aggressive management of TKI-induced cardiac toxicity and cardiovascular risk factors is advocated [30–32, 48], our study suggests in advanced cancer patients, given the overwhelming mortality from malignancy, aggressive cardiovascular risk factor management is unlikely to significantly impact survival. These results are consistent with recent randomized data showing that discontinuation of preventative cardiac medications does not lead to worse survival and may improve quality of life in metastatic patients [49].

The practice of aggressive cardiac risk factor management may have contributed to the low rates of cardiovascular events and cerebrovascular accidents in this study. Toxicity has been suggested as a pharmacodynamics marker for targeted anti-neoplastic drugs [50]. On-target side effects, such as arterial hypertension, can potentially serve as a biomarker for efficacy [51, 52]. Although a risk factor for cardio- and cerebrovascular disease, aggressive management with anti-hypertensive medications is advocated for as opposed to dose reduction or discontinuation [31, 32]. When cardiovascular side effects represent off-target side effects (which are not pharmacodynamics markers), dose maintenance is still advocated to maximize oncologic control through on-target mechanisms [31, 32].

Fortunately, most cardiovascular toxicities specific to TKIs appear reversible with aggressive and early management [13, 32, 48]. This reversibility is increasingly important in the management of subgroups of patients with good prognoses. Renal cell carcinoma, for example, can behave heterogeneously, with patients with good prognosis disease remaining on TKIs for several years [53]. In such subgroups of patients, particularly if cardiac risk factors exist, aggressive cardiac optimization may prevent deaths from cardiovascular outcomes. As many of the events in this study occurred later in the follow-up period, this finding is applicable to individuals who remain on TKIs for long periods of time. TKI usage is also being increasingly used in the adjuvant setting for prolonged durations [54], thus further necessitating minimization of long-term adverse effects.

Other known baseline cardiovascular risk factors may have contributed to death events, yet may not have been adequately captured. Risk factors such as diabetes and renal disease [55, 56] were not included in the validated algorithm used to establish baseline IHD [46]. Those with baseline diabetes or renal disease may not have been as aggressively managed for these comorbidities. Often, concerns of hypoglycaemia result in relaxed glycemic control [57, 58]. Those without overt baseline IHD, yet with other cardiovascular risk factors may be at higher likelihood for adverse events. Due to small numbers, a subgroup analysis to assess this was not possible.

The validated IHD algorithm used in this study assessed two physician billing codes (with one of the billing codes being from a physician in a hospital or emergency room setting) or one hospital discharge abstract to identify patients with IHD [46]. This algorithm excluded family physician diagnosis of angina or silent MI, which can be exclusively managed as an outpatient, and may have led to under-reporting of the prevalence of IHD [46].

This study has limitations. First, there is the potential for selection bias. Medications included in our analysis were available only to patients meeting certain criteria consistent with the registration trial supporting marketing of this drug. The effect of these drugs on individuals not meeting these criteria is unclear. Additionally, the NDFP only captures new or more expensive systemic treatments administered throughout the province. The proportion of cancer patients receiving chemotherapy prior to TKI initiation may be higher than captured through this study. The impact on subsequent cardiac outcomes is unlikely given the currently high proportion of chemotherapy use identified. Also, as a passive cancer registry, the OCR may not identify all cancer cases, as non-registry personnel may not be familiar with all reporting criteria and terminology. Second, we were unable to capture individuals treated with TKIs funded by mechanisms other than ODB, such as private drug insurance and self-pay options. Individuals with private drug coverage may also represent a different distribution of socioeconomic status (SES) compared to those relying on public healthcare coverage. SES has been shown to impact the survival of oncology patients, with lower SES being linked to poorer outcomes and decreased survival [59–61]. Thirdly, as a population-based study, expansion of the sample size to increase event rates is not possible. Finally, granularity of detail is not available in the ICES administrative databases. Therefore, information relating to previous radiation treatment, dose of TKI, duration of treatment and cause of death was not available in most cases. This limitation, may lead to some uncertainty regarding the results.

In summary, individuals treated with TKIs have a significantly higher hazard of death relative to the general population. Cause specific hazards of IHD and of cerebrovascular accidents are not increased. Our results are consistent with recent randomized data suggesting that discontinuation of cardio-protective medications is safe, presumably since the absolute rate of cardiac events is very low. The increased mortality identified in this study is likely reflective of the underlying malignant process. More careful surveillance and management of cardiac risks is likely only warranted in the subgroup of patients with an expected prolonged survival.
